# Follow-Up and Management of Chronic Rhinosinusitis in Adults with Primary Ciliary Dyskinesia: Review and Experience of Our Reference Centers

**DOI:** 10.3390/jcm8091495

**Published:** 2019-09-19

**Authors:** Emilie Bequignon, Laurence Dupuy, Virginie Escabasse, Francoise Zerah-Lancner, Laurence Bassinet, Isabelle Honoré, Marie Legendre, Marie Devars du Mayne, Bruno Crestani, Estelle Escudier, André Coste, Jean-François Papon, Bernard Maître

**Affiliations:** 1 Public Hospital Network of Paris (AP-HP), Henri Mondor Hospital, Intercommunal Hospital of Creteil Department of Otorhinolaryngology, 94010 Créteil, France; laurencedupuy@hotmail.fr (L.D.); Virginie.Escabasse@chicreteil.fr (V.E.); francoise.zerah@aphp.fr (F.Z.-L.); mariedevars@gmail.com (M.D.d.M.); Andre.Coste@chicreteil.fr (A.C.); 2 National Institute of Health and Medical Research INSERM, U955, Mondor Institute of biomedical research (IMRB), U955, 94010 Créteil, France; jean-francois.papon@aphp.fr (J.-F.P.); bm.maitre@gmail.com (B.M.); 3 Faculty of Medicine, Paris East University, F-94010 Créteil, France; 4 The National Center for Scientific Research CNRS, ERL 7000, 94010 Créteil, France; 5 Public Hospital Network of Paris (AP-HP), Henri Mondor Hospital Department of Physiology and Functional Explorations, 94010 Créteil, France; 6 Intercommunal Hospital of Creteil, Department of pneumology, 94010 Créteil, France; kilad@orange.fr; 7 Public Hospital Network of Paris (AP-HP), Cochin Hospital, Department of pneumology, 75014 Paris, France; isabelle.honore2@aphp.fr; 8 Faculty of Medicine, Paris Descartes University, 75014 Paris, France; 9 Public Hospital Network of Paris (AP-HP), Department of Embryology and Genetics Armand-Trousseau Hospital, 75012 Paris, France; marie.legendre@aphp.fr (M.L.);; 10 National Institute of Health and Medical Research, U933, Pierre and Marie Curie University, 75005 Paris, France; 11 National Institute of Health and Medical Research, U1152, 75018 Paris, France; bruno.crestani@aphp.fr; 12 University Department (DHU) Fibrosis, Inflammation and Remodeling in Renal and Respiratory Diseases (FIRE), 75018 Paris, France; 13 LabEx Inflamex, 75018 Paris, France; 14 Faculty of Medicine, Paris Diderot University, 75018 Paris, France; 15 Public Hospital Network of Paris (AP-HP), Bichat Hospital, Department of Pneumology, 75018 Paris, France; 16 Public Hospital Network of Paris (AP-HP), Kremlin Bicêtre Hospital, Department of Otorhinolaryngology, 94275 Le Kremlin-Bicêtre, France; 17 Faculty of Medicine, Paris South University, F-94070 Kremlin-Bicêtre, France

**Keywords:** primary ciliary dyskinesia, chronic rhinosinusitis, *Pseudomonas aeruginosa*

## Abstract

Chronic rhinosinusitis is the foremost manifestation in adult patients with primary ciliary dyskinesia (PCD). We present a retrospective series of 41 adult patients with a confirmed diagnosis of PCD followed in our reference centers. As part of the diagnostic work up in our centers, sinus computed tomography scans (CTs) are systematically performed. All patients also undergo a sampling of purulent secretions sampled from the middle meatus under endoscopic view for bacteriological analysis. In our series, CT opacities were consistent in all the patients, as well as mainly partial and located in ethmoid cells (100% of patients) and in maxillary sinuses (85.4% of patients), and stayed stable over time. In the 31 patients who had purulent secretions, bacteriological culture showed at least one bacterium in 83.9% (*n* = 26). There was no significant difference in positive cultures for *Pseudomonas aeruginosa* in patients >40 years old versus those <40 (*p* = 0.17; Fisher). Surgical management was performed in only 19% of patients in order to improve sinonasal mechanical drainage. Our data support the hypothesis that the sinuses can be considered as a bacterial reservoir. From this retrospective study, we have introduced several changes into our routine clinical practice in our reference centers. Based on our analyses, medical and surgical treatments benefit from incorporating bacteriological information and sinonasal symptoms much more than CT scan evaluation alone. All patients now undergo systematically an annual simultaneous bacteriological sampling of the middle meatus and sputum to follow the relationship between ENT and lung disease and to help to antibiotic therapy strategy.

## 1. Introduction

Primary ciliary dyskinesia (PCD) is an autosomal recessive genetic disorder leading to structural and/or functional abnormalities of the cilia. The resulting poor airway mucociliary clearance is responsible for recurrent infections of the upper and lower airways, the symptoms of which are typically present from birth. *Situs inversus* is found in nearly half of the patients, and fertility disorders are frequent [1]. Diagnosis is based on the association of clinical sinopulmonary syndrome with early onset in childhood coupled with specialized tests: nasal nitric oxide concentrations (NO); analysis of ciliary beat pattern by high-speed videomicroscopy and/or analysis of cilia ultrastructure by electronic microscopy; and/or identification of an unambiguous causal mutation in a known PCD gene. In a recent state-of-the-art study [2], Honoré et al. underlined the fact that the management of PCD remains poorly defined and should be performed by specialized centers in order to standardize follow-up and improve patient outcome. Chronic rhinosinusitis (CRS) in patients with PCD is at the forefront of the disease presentation in adults [3], but it has been poorly studied [4]. The aim of this review was to compare the importance of bacteriological data to better inform the treatment and management of PCD patients with CRS.

## 2. Methods

We reported the bacteriological and radiological characteristics and management of CRS in a series of 41 adults with PCD managed in our reference centers (members of the French National Reference Centre for Rare Respiratory Diseases (RESPIRARE)), and compared our findings with the literature. The National Centre’s Ethics Committee approved the retrospective use of the register (CCTIRS, no.08.015bis). Data from computed tomography (CT) scans of paranasal and sinus microbiological analysis (when available) were analyzed. We used Fisher’s exact test to study the correlation between the presence of a positive culture for *Pseudomonas aeruginosa* (*P. aeruginosa*) and age (over 40 years old versus below 40).

## 3. Results

### 3.1. Clinical Evaluation of Chronic Rhinosinusitis in Adults with PCD

CRS is a major problem in adults with PCD [3], while children mainly present otologic disease with binaural otitis media and effusion (OME), leading to hearing impairment [5]. However, some authors report non-specific rhinological symptoms in children with PCD, such as daily nasal discharge and congestion (i.e., rhinitis) without remission starting soon after birth [3] and episodic facial pain [6,7]. Nasal polyps are rarely described in childhood [7]. Sinusitis was reported in 37% of a small series of 17 children with PCD (mean age of 6.5 years) [8]. Severe CRS is reported in older children [6,7]. In adults with PCD, an atypical chronic edematous and purulent sinusitis is frequently described, with anosmia-like associated symptoms [9]. We have already published that CRS is present in 94.8% of adult patients with PCD, and nasal polyps are reported in 15% to 56% [1,2]. In a series of 110 patients with PCD (ranging from the neonatal period up to late adulthood (mean age, 26.8, range 0–73), all subjects had a history of chronic upper airway symptoms/chronic rhinitis [10]. In our Ear, Nose and Throat (ENT) centers, a standardized evaluation is performed by a senior expert in rhinology. Nasal symptoms (i.e., rhinorrhoea, nasal obstruction, facial pain, dysosmia, sneezing, epistaxis) are scored by means of a semi-quantitative scale from 0 to 3 for each symptom (0: absent, 1: mild, 2: moderate, and 3: severe) giving an overall maximum score of 18 [11]. An endoscopic examination of the nose is systematically performed in order to evaluate the aspect of mucosa, secretions, and the presence of turbinate swelling. Associated factors of nasal obstruction (i.e., external valve abnormality, septal deviation, swelling of the turbinates) and the effectiveness of nasal irrigation are systematically reported. Furthermore, for the last three years, we have used a nasal endoscopic score (out of 10) at each follow-up visit (Table 1). It was already shown that daily pulmonary symptoms such as cough and shortness of breath and pulmonary exacerbations significantly impact quality of life of these patients [12,13,14]. Since the validation of the health-related quality of life instrument for PCD (QOL-PCD) [15], QOL-PCD was systematically used to evaluate the quality of life of patients at each follow-up visit. A specific evaluation of quality life focusing on nasal symptoms: Sinonasal Outcome Test 22 (SNOT-22) is now also performed in our centers in order to evaluate the impact of our treatments for rhinosinusitis.

### 3.2. Computed Tomography Evaluation of Chronic Rhinosinusitis

Hypoplasia and agenesis of the paranasal sinuses (mainly frontal sinuses) is found in more than two-thirds of patients with PCD [16]. Accordingly, sinus hypoplasia or aplasia was identified in 58% of the patients in a recent series of 31 PCD patients who underwent endoscopic surgery [17]. In our ENT centers, sinus CT is systematically performed on the confirmation of PCD to assess the severity of the disease (opacities and bone remodeling) and the presence of associated obstructive patterns (septal deviation, concha bullosa, and Haller cells) or complications (mucocele, pyocele, and bone lysis). In our series of 41 patients, CT opacities were consistent in all the patients and mainly found in the ethmoid cells (100% of patients) and maxillary sinuses (85.4% of patients). Hypoplasia and agenesis mainly involve the frontal or sphenoidal sinuses. All the sinus CT characteristics of our series are listed in Table 2. Initially, CT follow-up was performed in our series, but no complication such as mucocele was observed, and opacities were mainly partial and stable over time, whatever the management and the course of symptoms. Thus, sinus CT is not performed anymore in follow-up, but can be useful in case surgery is planned.

### 3.3. Microbiological Analysis in Chronic Rhinosinusitis in Adults with PCD

In our ENT centers, aspiration of the middle meatus was performed when purulent secretions were seen under endoscopy, and the samples undergo bacteriological and mycological analysis. In our series, a microbiological analysis was performed in 31 of the 41 patients, and showed the presence of at least one bacterium in 83.9% (*n* = 26). No yeast or fungus was found. These bacteriological findings are in line with Alanin et al.’s recent review. Table 3 lists all the microorganisms found in our series. We did not find any significant difference in the prevalence of positive cultures for *P. aeruginosa* in patients over 40 years old versus those below 40 (*p* = 0.17; Fisher). Our findings support the hypothesis that the sinuses can be considered as a bacterial reservoir and could be a target for surgical and antibiotic treatment in patients with PCD [18]. Thus, in our reference centers, all patients now undergo systematically an annual simultaneous bacteriological sampling of the middle meatus and sputum to follow the relationship between ENT and lung disease, and to help to antibiotic therapy strategy. An ongoing prospective study is performed in the hospital of Creteil (ClinicalTrials.gov Identifier: NCT03494894), which is called the “Bacteriological Link Between Upper and Lower Airways in Cystic Fibrosis and Primary Ciliary dyskinesia”.

A recent review of sinus bacteriology in patients with PCD reported that *Haemophilus influenzae* (*H. influenzae*) is the most common bacteria (28%), followed by *Streptococcus pneumoniae* (*S. pneumoniae*) and *P. aeruginosa* [19]. However, when nonsurgical swabs and blowing samples are excluded, *P. aeruginosa* emerges as the most common sinus bacteria in patients with PCD (50%), followed by *Staphylococcus aureus* (*S. aureus*) and *H. influenzae* [19]. Along the same lines, the most common pathogens found in bronchial secretion cultures in adults with PCD are *H. influenzae*, *S. aureus*, and *P. aeruginosa* [2]. In Noone et al.’s retrospective series, *P. aeruginosa* was also found to be common in the lower airways, even at a young age (5 years), although in 12 of the subjects, the appearance of *P. aeruginosa* was delayed, first appearing after age 30 in most (11/12 patients of positive samples) [10]. According to this author, chronic *P. aeruginosa* lung colonization could be a prognostic factor in patients with PCD [10]. Indeed, PCD patients with *P. aeruginosa* colonization of the lower airways have significantly poorer respiratory function than other patients [1]. In a recent prospective analysis, Alanin et al. found that the prevalence of *P. aeruginosa* in the sinus increased with age [20]. In a recent study of seven PCD patients who underwent functional endoscopic sinus surgery, five (71%) had the same bacteria in the sinus and lung cultures: *P. aeruginosa* in four of the five (80%), and *H. influenzae* in one (20%) [18]. The most striking argument sustaining this hypothesis is lung re-colonization from the sinuses, which is frequently observed in lung-transplanted PCD patients [17]. These last years, various research groups have focused on exploring the microbiome to understand the role of bacteria in chronic rhinosinusitis. Koeller et al. found that the bacterial community of patients with chronic rhinosinusitis without nasal polyps differed significantly from the healthy controls [21]. In patients with cystic fibrosis, recent studies of gut microbiota also suggested that the disordered bacterial ecology of the cystic fibrosis gastrointestinal tract could be associated with pulmonary outcomes [22]. The association between decreased bacterial community diversity in the cystic fibrosis airway and lung disease progression was already shown, but the causal relationship between these phenomena has yet to be fully elucidated [23]. We can compare the complexity of the polymicrobial community in the upper airways of PCD patients with the cystic fibrosis lung, which is a very similar system. Some authors underlined how the biochemistry of cystic fibrosis lung could explain the composition and physiology of polymicrobiobial communities [24]. For example, Quinn et al. showed how microaerobic respiration by *P. aeruginosa* may occur as oxygen is depleted deeper in the mucus of cystic fibrosis lung, and then anaerobic metabolism via denitrification [24]. Amino acid metabolism was increased by *P. aeruginosa*, which induce after amino acid degradation a high level of ammonia in cystic fibrosis sputum, which can influence the sputum pH [24]. Based on these studies, further studies are needed to investigate the relation between bacterial community characteristics and the development of CRS in adults with PCD, and the relation between the microbiome of the upper airway and pulmonary outcomes. Moreover, further studies are needed to precisely determine the relationship between the initial evaluation and symptomatic, endoscopic, imaging, and bacteriological prognosis. A national study is currently performed to determine prognosis factors (symptomatic, endoscopic, imaging, and bacteriological) at initial evaluation, which is entitled the primary ciliary dyskinesia (PCD) national cohort: Identification of severity criteria from deep phenotyping and phenotype-genotype correlation study (ANR-lO-COHO-03-0) (RADICO (Rare Disease Cohorts) National program).

### 3.4. Management of Chronic Rhinosinusitis in Adults with PCD

To date, no results of controlled trials of treatment in PCD patients have been published. All recommendations are currently derived from cystic fibrosis guidelines and personal experiences without evidence of benefit [6,25]. The overall management of the patient’s disease must be based on consultation between pulmonologists and ENT specialists [2].

In the literature, medical treatment for CRS is based on nasal irrigation with isotonic or hypertonic serum, local corticosteroid therapy, and local (nebulization) and general antibiotic therapy [3]. In our reference centers, nasal irrigation is systematically prescribed twice a day for all patients as soon as PCD is diagnosed. A nurse specialized in therapeutic education explains the manual and instrumental techniques according to the patient’s choice (syringe, serum reservoir with adapted nasal insert, position of the head). Nasal irrigations are mainly based on isotonic saline, but in case of severe obstruction, we now give hypertonic saline, based on the positive effect of nebulized hypertonic saline on the quality of life in adults with PCD [12].

In the case of purulent rhinosinusitis, in our centers, oral antibiotics were the main treatment, which was exclusively guided on nasal bacteriological analysis. In a recent Cochrane database review [26] about antibiotic strategies for eradicating *P. aeruginosa* in patients with cystic fibrosis, the authors found that nebulized antibiotics (inhaled tobramycin or colistin solution), alone or in combination with oral antibiotics (ciprofloxacin), were better than no treatment for early infection with *P. aeruginosa* [26]. However, the authors could not conclude whether antibiotic strategy should be used for the eradication of early *P. aeruginosa* because of insufficient evidence [26]. Similarly, there is a lack of randomized controlled trials investigating the efficacy of intravenous antibiotics to eradicate *P. aeruginosa* in PCD. According to the bacterial reservoir theory (as sinuses can be considered as a bacterial reservoir for the lower airways), we adapted our practices and administrate antibiotics via oral, intravenous, or nebulized ways, taking in account: (i) the combined bacterial analysis of the nose and the sputum; (ii) the bacterial resistance, which may requires intravenous antibiotics, and (iii) the distinction between the primary *P. aeruginosa* infection and *P. aeruginosa* colonization. In our centers, patients are treated with nebulized antibiotics when the sinus culture shows colonization with *P. aeruginosa* associated with an exacerbation of CRS, even with negative lung culture. Sonic aerosol therapies are used to target the maxillary sinuses rather than the lungs [27] according to a consensus statement for the prescription of nebulization in rhinology by the working group of the French Society of Otorhinolaryngology (SFORL) [28].

In case of rhinosinusitis with inflammatory polyps or mucosal oedema, local corticosteroids were prescribed in 73% of patients in our series (Table 4).

In the literature, the indication of long-term low-dose macrolide anti-inflammatory therapy remains to be evaluated in ENT indications, but may be a useful option in selected cases [2]. A double-blind placebo controlled trial of the effects of six months’ treatment with azithromycin on airway infection and lung function in children and adults with PCD has finished, and results are being analyzed, but are not yet published [29]. We adapted our practice by giving long-term low-dose macrolide anti-inflammatory therapy before surgery.

In our series, sinonasal surgical treatment was performed in 19% of the patients. All surgery procedures were described in Table 4. Indications of surgery were major nasal obstruction due to the hypertrophy of inferior turbinates (indication for turbinate reduction), facial pain due to blockage of the osteomeatal complex of maxillary or frontal sinus (indication for ethmoidectomy). In our series, patients who underwent ethmoidectomy had associated turbinate reduction (*n* = 4). In one patient, failure of ethmoidectomy induced nasofrontal duct stenosis and major frontal headache, which required the complete obliteration of the frontal sinus. Improvement of facial pain was observed after ethmoidectomy in all patients, while rhinorrhea was not improved. Turbinate reduction improved the sensation of nasal obstruction. Thus, we now guide our surgical indication based on predominant symptoms: if facial pain is at the forefront, an ethmoidectomy is performed, while in the case of major nasal obstruction, a turbinate reduction is performed under local anesthesia without ethmoidectomy to improve nasal ventilation.

In the literature, the long-term outcome of sinonasal surgery in PCD has not been studied to date. In Noone et al.’s series, previous sinus surgery was reported in 37% of patients (19 adults and 10 children) [10]. Only one prospective study has evaluated the surgical treatment of CRS in PCD [4,20]. Patients with lower airway obstructive disease must be preoperatively managed (chest physiotherapy for bronchial drainage, adapted antibiotics, smoking cessation) to limit the risks associated with general anesthesia with intubation. The post-operative period sometimes requires intensive care supervision. The objectives of sinonasal surgery in PCD patients are to: treat nasal obstruction to restore nasal breathing; improve olfaction and sinus mechanical drainage; and improve the diffusion of local therapies. Some retrospective studies also suggest that sinus surgery may reduce the respiratory bacterial reservoir [17]. In a retrospective study of three children with PCD, functional endoscopic sinus surgery resulted in a marked improvement in symptomatology with a decreased incidence of hospitalization and a decreased need for medical therapy [30]. In a recent prospective study of 31 adults with PCD, functional endoscopic sinus surgery with adjuvant therapy was shown to eradicate sinus bacteria, thereby reducing lung re-colonization from the sinuses [20]. In this study, post-operative adjuvant therapy was standardized, and included two weeks of systemic antibiotic therapy according to the susceptibility testing of the bacteria cultured from sinuses and twice-daily nasal irrigation with saline and topical nasal steroids (spray mometasonefuroate 100 milligrams) for at least three months [20]. The aim of post-surgical treatment is to maintain adequate drainage. Table 5 reports the newly implemented ENT clinical practices in adults with PCD and expectations of these changes.

## 4. Conclusions

Over the 20 last years, we have introduced several changes into our routine clinical practice. In our experience, three-quarters of patients had nasal purulent secretions with the presence of at least one bacterium in 84%, supporting the hypothesis that the sinuses can be considered as a bacterial reservoir. In order to better inform our patients about the treatment and management of their PCD with CRS, samples of the middle meatus and sputum are now taken annually from all patients for bacteriological analysis to follow the relationship between ENT and lung diseases. A sinus CT scan systematically performed at diagnosis for initial assessment of the disease showed abnormalities such as opacities and/or hypoplasia/agenesis in all patients. We no longer performed the CT scan as a follow-up exam, because no complication such as mucocele was found, and opacities stayed mainly partial and stable over time, whatever the management. In our experience, the rate of surgical management was low. Based on our analyses, medical and surgical treatments benefit from incorporating bacteriological information and sinonasal symptoms much more than CT scan evaluation alone. Expectations from these changes will be: reduced rates of infection, reduced antibiotic use, and reduced surgery use in order to improve the quality of life of patients. CRS in PCD adults requires standardized ENT evaluation and management in an expert center.

## Figures and Tables

**Table 1:** Standardized nasal endoscopic score in adults with primary ciliary dyskinesia (PCD).

Erythema	0 no
	1 yes
Oedema	0 no
	1 yes
Polyps	0 no
	1 yes
Purulent nasal secretions	0 no
	1 yes
Crusting	0 no
	1 yes
Total points: 10 (5 points for each side of the nasal fossa)	

**Table 2:** Sinus computed tomography (CT) characteristics (*n* = 41).

	Development Abnormalities		Opacities		Bone Thickening
	Agenesia n (%)	Hypoplasia n (%)	Incomplete n (%)	Complete n (%)	n (%)
Maxillary sinus	1 (2.4)	2 (4.9)	30 (73.2)	5 (12.2)	2 (4.9)
Anterior ethmoidal sinus	0 (0)	4 (9.8)	29 (70.7)	5 (12.2)	3 (7.3)
Posterior ethmoidal sinus	0 (0)	4 (9.8)	28 (68.3)	5 (12.2)	3 (7.3)
Frontal sinus	7 (17.1)	6 (14.6)	20 (48.8)	4 (9.8)	1 (2.4)
Sphenoidal sinus	0 (0)	10 (24.4)	19 (49.3)	2 (4.9)	3 (7.3)

**Table 3:** Middle meatus bacteriological analysis (*n* = 31).

Microorganism	Patient (*n*)	%
*Haemophilus influenzae*	8	25.8
*Streptoccocus pneumoniae*	6	19.3
*Pseudomonas aeruginosa*	6	19.3
*Staphylococcus aureus*	2	6.4
*Escherichia coli*	1	3.2
*Branhamella catarrhalis*	1	3.2
*Citrobacter koseri*	1	3.2
*Klebsielle oxytoca*	1	3.2
*Serratia marcencens*	1	3.2
*Corynebacterium*	1	3.2
*Moraxella catarrhalis*	1	3.2
*Propionibacterium acnes*	1	3.2
*Klebsielle pneumoniae*	1	3.2

**Table 4:** Medical and surgical management of PCD adults (*n* = 41) in our centers.

Management	Patient (*n*)	%
*Medical management*		
Nasal irrigation	41	100
Local corticosteroid therapy	30	73
Nebulized antibiotics	7	17
*Surgical management*	8	19.5
Turbinate reduction	7	17
Ethmoidectomy	4	9.8
Frontal sinus obliteration	1	2.4

**Table 5:** Experience of our centers: newly implemented ENT clinical practices in adults with PCD and expectations of these changes.

	Past ENT Practices	New ENT Practices	Expectations
Initial evaluation	Personal history	Personal history with nasal symptom score	Evaluation improvement
	Nasal endoscopy		
	CT scan	Nasal endoscopy with endoscopic score	
		Sinus CT scan	
		Nasal and sputum bacteriological analysis	
Management			
Inferior turbinate hypertrophy and severe nasal obstruction	Nasal irrigation with isotonic saline	Nasal irrigation with isotonic or hypertonic saline	Quality of life improvement
	Ethmoidectomy with turbinate reduction under general anesthesia	Turbinate reduction under local anesthesia	Mini-invasive surgery
Purulent rhinosinusitis	Oral antibiotics based on nasal bacteriology	Oral or intravenous or nebulized antibiotics based on:	Reduced antibiotic use
		Combined bacterial results of the nose and sputum	Improvement of antibiotic efficiency
		Resistance profile	
		Evidence of pseudomonas Aeruginosa colonization	
		Ethmoidectomy in case of severe facial pain	
Rhinosinusitis with polyps	Local corticosteroids	Local corticosteroids	Improvement of medical treatment
		Long-term low-dose macrolide	
		Ethmoidectomy in case of severe nasal obstruction or facial pain	Improvement of patient selection for surgery
Follow-up	Nasal endoscopy	Nasal symptom score	Reduce CT irradiation
	CT scan	Nasal endoscopic score	Improvement of evaluation
		Nasal and sputum bacteriological analysis	
